# Tris[*N*-(2-furoyl)-*N*,*N*′-diphenyl­thio­ureato-κ^2^ *O*,*S*]cobalt(III)

**DOI:** 10.1107/S1600536808011598

**Published:** 2008-04-30

**Authors:** Hiram Pérez, Rodrigo S. Corrêa, Ana María Plutín, Beatriz O’Reilly, Julio Duque

**Affiliations:** a Departamento de Química Inorgánica, Facultad de Química, Universidad de la Habana, Habana 10400, Cuba; b Grupo de Cristalografía, Instituto de Física de São Carlos, Universidade de São Paulo, São Carlos, Brazil; c Laboratorio de Síntesis Orgánica, Facultad de Química, Universidad de la Habana, Habana 10400, Cuba; d Instituto de Ciencia y Tecnología de Materiales, Universidad de la Habana, Habana 10400, Cuba

## Abstract

In the title compound, [Co(C_18_H_13_N_2_O_2_S)_3_], the Co^III^ atom is coordinated by the S and O atoms of three *N*-furoyl-*N*′,*N*′-diphenyl­thio­urea ligands in a slightly distorted octa­hedral geometry. The three O atoms are arranged *fac*, as are the three S atoms.

## Related literature

For general background, see: Arslan *et al.* (2003 ▶). For related structures, see: Jia *et al.* (2007 ▶); Pérez *et al.* (2008 ▶). For the synthesis of the ligand, see: Hernández *et al.* (2003 ▶).
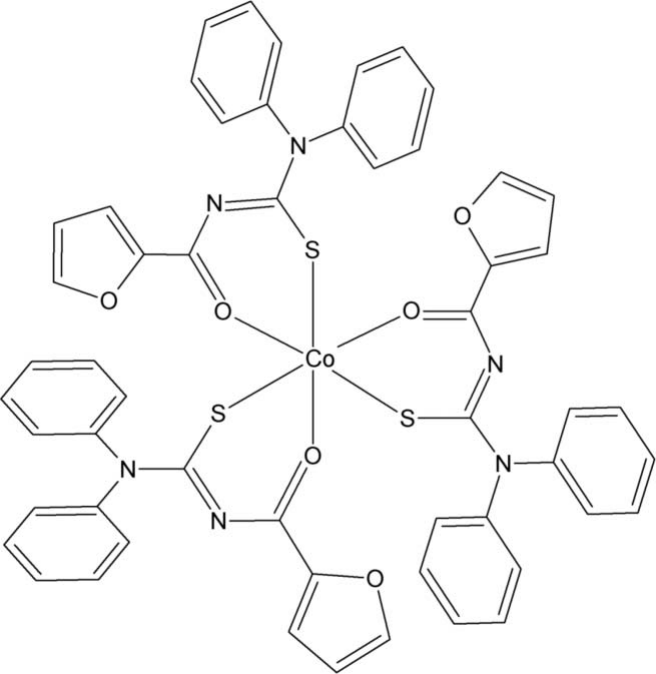


## Experimental

### 

#### Crystal data

[Co(C_18_H_13_N_2_O_2_S)_3_]
*M* *_r_* = 1023.05
Triclinic, 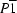

*a* = 10.0236 (11) Å
*b* = 13.1438 (16) Å
*c* = 19.388 (3) Å
α = 79.357 (7)°
β = 83.477 (8)°
γ = 71.152 (8)°
*V* = 2371.8 (5) Å^3^
*Z* = 2
Mo *K*α radiation
μ = 0.55 mm^−1^
*T* = 150 (2) K
0.15 × 0.13 × 0.02 mm

#### Data collection

Nonius KappaCCD diffractometer
Absorption correction: Gaussian (Coppens *et al.*, 1965 ▶) *T*_min_ = 0.955, *T*_max_ = 0.980
13609 measured reflections
8289 independent reflections
4680 reflections with *I* > 2σ(*I*)
*R*_int_ = 0.092

#### Refinement

*R*[*F* ^2^ > 2σ(*F* ^2^)] = 0.086
*wR*(*F* ^2^) = 0.222
*S* = 1.19
8289 reflections
632 parameters
H-atom parameters constrained
Δρ_max_ = 0.51 e Å^−3^
Δρ_min_ = −0.53 e Å^−3^

### 

Data collection: *COLLECT* (Nonius, 1998 ▶); cell refinement: *DENZO*/*SCALEPACK* (Otwinowski & Minor, 1997 ▶); data reduction: *DENZO*/*SCALEPACK*; program(s) used to solve structure: *SHELXS97* (Sheldrick, 2008 ▶); program(s) used to refine structure: *SHELXL97* (Sheldrick, 2008 ▶); molecular graphics: *ORTEP-3* (Farrugia, 1997 ▶); software used to prepare material for publication: *WinGX* (Farrugia, 1999 ▶).

## Supplementary Material

Crystal structure: contains datablocks global, I. DOI: 10.1107/S1600536808011598/hy2130sup1.cif

Structure factors: contains datablocks I. DOI: 10.1107/S1600536808011598/hy2130Isup2.hkl

Additional supplementary materials:  crystallographic information; 3D view; checkCIF report

## Figures and Tables

**Table:** 

O1—Co1	1.939 (5)
O3—Co1	1.920 (5)
O5—Co1	1.919 (4)
S1—Co1	2.217 (2)
S2—Co1	2.214 (2)
S3—Co1	2.196 (2)

**Table:** 

O5—Co1—O3	88.20 (19)
O5—Co1—O1	85.77 (19)
O3—Co1—O1	85.5 (2)
O5—Co1—S3	93.19 (15)
O3—Co1—S3	176.11 (15)
O1—Co1—S3	90.95 (15)
O5—Co1—S2	91.05 (15)
O3—Co1—S2	93.98 (15)
O1—Co1—S2	176.80 (14)
S3—Co1—S2	89.62 (8)
O5—Co1—S1	178.62 (16)
O3—Co1—S1	90.58 (13)
O1—Co1—S1	93.51 (14)
S3—Co1—S1	87.99 (7)
S2—Co1—S1	89.66 (7)

**Fig. 1.**
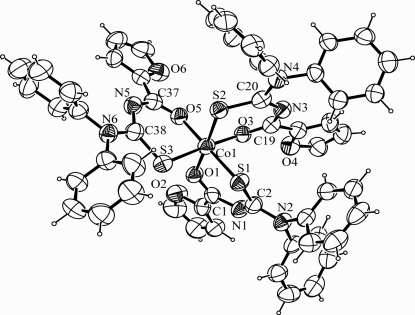
The molecular structure of the title compound. Displacement ellipsoids are drawn at the 50% probability level.

**Fig. 2.**
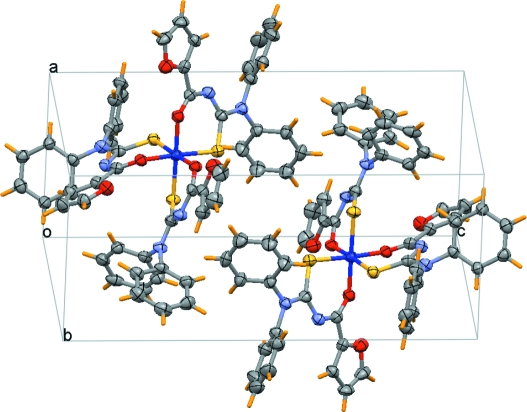
View of the unit cell of the title complex.

## supplementary crystallographic information

### Comment

Substituted *N*-acylthioureas are well known as chelating agents. Over recent years, many transition metal complexes with thiourea derivatives have been reported (Arslan *et al.*, 2003), because this kind of ligands display a remarkably rich coordination chemistry.

In this paper, we report the crystal structure of the title compound (Fig. 1), which presents an octahedral environment about the Co^III^ atom with the ligands coordinating in a relatively distorted manner (Table 1). The Co—S bond lengths lie within the range of those found in the related structure (Jia *et al.*, 2007; Pérez *et al.*, 2008). The lengths of C—O, C—S and C—N bonds in the chelate rings are between characteristic single and double bond lengths; they are shorter than single bond and longer than double bond. These results can be explained by the existence of delocalization in the chelate rings. Fig. 2 shows the arrangement of the complex molecules in the unit cell.

### Experimental

*N*-furoyl-*N*',*N*'-diphenylthiourea ligand was synthesized according to a procedure described by Hernández *et al.* (2003), by converting furoyl chloride into furoyl isothiocyanate and then condensing with an appropriate amine. To an ethanol solution (30 ml) containing the ligand (0.96 g, 3 mmol) was added an ethanol solution of Co(CH_3_COO)_2_.4H_2_O (0.25 g, 1 mmol). The solution was stirred at room temperature for 2 h, and at once a solution of NaOH (1 *N*) was added to adjust pH to the neutral value. The mixture was filtered and the filtrate was evaporated under reduced pressure to give a green solid, which was washed with acetone. Single crystals were obtained by slow evaporation of a chloroform/*N*,*N*-diphenylformamide solution (1:1, *v*/*v*) of the complex.

### Refinement

H atoms were positioned geometrically and refined as riding atoms, with C—H = 0.93 Å and *U*_iso_(H) = 1.2*U*_eq_(C).

### Crystal data

**Table:**

[Co(C_18_H_13_N_2_O_2_S)_3_]	*Z* = 2
*M**_r_* = 1023.05	*F*_000_ = 1056
Triclinic, *P*1	*D*_x_ = 1.432 Mg m^−^^3^
Hall symbol: -P 1	Mo *K*α radiation λ = 0.71073 Å
*a* = 10.0236 (11) Å	Cell parameters from 8767 reflections
*b* = 13.1438 (16) Å	θ = 2.9–25.4º
*c* = 19.388 (3) Å	µ = 0.55 mm^−^^1^
α = 79.357 (7)º	*T* = 150 (2) K
β = 83.477 (8)º	Block, green
γ = 71.152 (8)º	0.15 × 0.13 × 0.02 mm
*V* = 2371.8 (5) Å^3^	

### Data collection

**Table:**

Nonius KappaCCD diffractometer	4680 reflections with *I* > 2σ(*I*)
*T* = 150(2) K	*R*_int_ = 0.092
φ and ω scans	θ_max_ = 25º
Absorption correction: Gaussian(Coppens *et al.*, 1965)	θ_min_ = 3.0º
*T*_min_ = 0.955, *T*_max_ = 0.980	*h* = −11→11
13609 measured reflections	*k* = −15→14
8289 independent reflections	*l* = −23→23

### Refinement

**Table:**

Refinement on *F*^2^	H-atom parameters constrained
Least-squares matrix: full	*w* = 1/[σ^2^(*F*_o_^2^) + (0.069*P*)^2^ + 0.4724*P*] where *P* = (*F*_o_^2^ + 2*F*_c_^2^)/3
*R*[*F*^2^ > 2σ(*F*^2^)] = 0.086	(Δ/σ)_max_ < 0.001
*wR*(*F*^2^) = 0.222	Δρ_max_ = 0.51 e Å^−^^3^
*S* = 1.19	Δρ_min_ = −0.53 e Å^−^^3^
8289 reflections	Extinction correction: SHELXL97 (Sheldrick, 2008), Fc^*^=kFc[1+0.001xFc^2^λ^3^/sin(2θ)]^-1/4^
632 parameters	Extinction coefficient: 0.0076 (14)

### Special details

**Table:**

Geometry. All e.s.d.'s (except the e.s.d. in the dihedral angle between two l.s. planes) are estimated using the full covariance matrix. The cell e.s.d.'s are taken into account individually in the estimation of e.s.d.'s in distances, angles and torsion angles; correlations between e.s.d.'s in cell parameters are only used when they are defined by crystal symmetry. An approximate (isotropic) treatment of cell e.s.d.'s is used for estimating e.s.d.'s involving l.s. planes.

### Fractional atomic coordinates and isotropic or equivalent isotropic displacement parameters (Å^2^)

**Table:**

	*x*	*y*	*z*	*U*_iso_*/*U*_eq_	
C1	0.8134 (7)	0.8428 (6)	0.3061 (3)	0.0522 (17)	
C2	0.6159 (7)	0.8246 (6)	0.2602 (4)	0.0555 (18)	
C3	0.8605 (7)	0.9270 (6)	0.3268 (4)	0.0588 (19)	
C4	0.7991 (8)	1.0351 (6)	0.3228 (4)	0.066 (2)	
H4	0.7102	1.0748	0.3068	0.079*	
C5	0.8942 (10)	1.0766 (8)	0.3472 (5)	0.089 (3)	
H5	0.8798	1.149	0.3508	0.106*	
C6	1.0102 (10)	0.9925 (8)	0.3645 (5)	0.087 (3)	
H6	1.0903	0.9976	0.3818	0.104*	
C7	0.4392 (7)	1.0014 (5)	0.2305 (4)	0.0562 (19)	
C8	0.3801 (7)	1.0434 (6)	0.2904 (5)	0.068 (2)	
H8	0.3778	0.9973	0.3328	0.082*	
C9	0.3241 (8)	1.1545 (7)	0.2874 (5)	0.073 (2)	
H9	0.2838	1.1836	0.3279	0.087*	
C10	0.3278 (9)	1.2218 (7)	0.2253 (6)	0.082 (3)	
H10	0.2893	1.2969	0.223	0.099*	
C11	0.3895 (9)	1.1776 (7)	0.1656 (5)	0.076 (2)	
H11	0.3928	1.2235	0.1231	0.091*	
C12	0.4462 (8)	1.0663 (6)	0.1680 (4)	0.062 (2)	
H12	0.488	1.0367	0.1278	0.075*	
C13	0.4055 (7)	0.8386 (5)	0.1994 (4)	0.0556 (18)	
C14	0.2771 (7)	0.8351 (6)	0.2318 (4)	0.064 (2)	
H14	0.2509	0.8556	0.2761	0.076*	
C15	0.1880 (8)	0.8017 (7)	0.1994 (5)	0.077 (2)	
H15	0.1024	0.7974	0.2218	0.093*	
C16	0.2263 (8)	0.7744 (6)	0.1329 (5)	0.073 (2)	
H16	0.1659	0.7529	0.1099	0.087*	
C17	0.3530 (8)	0.7791 (6)	0.1013 (4)	0.067 (2)	
H17	0.3786	0.7603	0.0566	0.08*	
C18	0.4443 (7)	0.8113 (6)	0.1343 (4)	0.0613 (19)	
H18	0.5307	0.8141	0.1123	0.074*	
C19	0.8633 (7)	0.7096 (5)	0.1301 (4)	0.0530 (17)	
C20	0.8537 (6)	0.5288 (5)	0.1438 (4)	0.0521 (17)	
C21	0.8372 (7)	0.8086 (5)	0.0784 (4)	0.0529 (17)	
C22	0.7907 (8)	0.8320 (6)	0.0137 (4)	0.064 (2)	
H22	0.7667	0.7857	−0.0101	0.077*	
C23	0.7852 (9)	0.9420 (6)	−0.0116 (5)	0.076 (2)	
H23	0.7561	0.9819	−0.0551	0.091*	
C24	0.8294 (8)	0.9762 (7)	0.0388 (4)	0.070 (2)	
H24	0.8379	1.0454	0.0355	0.084*	
C25	0.7778 (7)	0.4928 (6)	0.0372 (4)	0.0527 (17)	
C26	0.6395 (7)	0.5639 (6)	0.0319 (4)	0.065 (2)	
H26	0.588	0.5922	0.0708	0.078*	
C27	0.5821 (8)	0.5905 (7)	−0.0333 (4)	0.066 (2)	
H27	0.4905	0.6376	−0.038	0.079*	
C28	0.6569 (8)	0.5492 (6)	−0.0910 (4)	0.067 (2)	
H28	0.6169	0.5683	−0.1344	0.081*	
C29	0.7912 (7)	0.4796 (6)	−0.0840 (4)	0.0608 (19)	
H29	0.842	0.4503	−0.1227	0.073*	
C30	0.8521 (7)	0.4524 (6)	−0.0202 (4)	0.0576 (18)	
H30	0.9443	0.4062	−0.0163	0.069*	
C31	0.8687 (7)	0.3430 (5)	0.1307 (4)	0.0546 (18)	
C32	0.7627 (8)	0.2952 (6)	0.1373 (4)	0.0598 (19)	
H32	0.67	0.3382	0.1298	0.072*	
C33	0.7944 (8)	0.1840 (6)	0.1550 (4)	0.069 (2)	
H33	0.7231	0.1521	0.1581	0.083*	
C34	0.9307 (9)	0.1195 (6)	0.1682 (4)	0.068 (2)	
H34	0.9513	0.0445	0.1806	0.081*	
C35	1.0364 (8)	0.1675 (6)	0.1628 (4)	0.067 (2)	
H35	1.1283	0.1248	0.1724	0.081*	
C36	1.0056 (7)	0.2786 (6)	0.1432 (4)	0.0593 (19)	
H36	1.0775	0.3103	0.1383	0.071*	
C37	1.1649 (7)	0.4839 (6)	0.3130 (4)	0.0509 (17)	
C38	1.0025 (7)	0.4247 (6)	0.3945 (4)	0.0541 (18)	
C39	1.3184 (7)	0.4579 (6)	0.2980 (4)	0.0518 (17)	
C40	1.4255 (8)	0.3729 (6)	0.3245 (4)	0.0600 (19)	
H40	1.4202	0.3139	0.3582	0.072*	
C41	1.5501 (8)	0.3934 (8)	0.2891 (5)	0.080 (3)	
H41	1.6426	0.3489	0.2953	0.096*	
C42	1.5094 (8)	0.4877 (8)	0.2457 (5)	0.077 (3)	
H42	1.57	0.52	0.2168	0.093*	
C43	0.8608 (7)	0.3438 (6)	0.4858 (4)	0.0571 (18)	
C44	0.8498 (8)	0.3463 (6)	0.5573 (4)	0.062 (2)	
H44	0.9256	0.3484	0.5799	0.075*	
C45	0.7243 (9)	0.3454 (6)	0.5943 (4)	0.070 (2)	
H45	0.7162	0.3463	0.6425	0.084*	
C46	0.6115 (9)	0.3433 (7)	0.5617 (5)	0.080 (2)	
H46	0.5271	0.344	0.5873	0.096*	
C47	0.6237 (9)	0.3400 (8)	0.4914 (5)	0.088 (3)	
H47	0.547	0.3383	0.4693	0.105*	
C48	0.7462 (8)	0.3392 (7)	0.4529 (4)	0.079 (2)	
H48	0.7537	0.3355	0.4051	0.095*	
C49	1.1159 (7)	0.2534 (6)	0.4715 (4)	0.0565 (18)	
C50	1.2270 (8)	0.2720 (7)	0.4956 (4)	0.069 (2)	
H50	1.2286	0.3428	0.494	0.083*	
C51	1.3385 (9)	0.1833 (9)	0.5227 (5)	0.087 (3)	
H51	1.4146	0.1948	0.5398	0.104*	
C52	1.3359 (11)	0.0805 (9)	0.5241 (5)	0.099 (3)	
H52	1.4103	0.0222	0.5429	0.119*	
C53	1.2261 (12)	0.0598 (8)	0.4984 (5)	0.099 (3)	
H53	1.2264	−0.0111	0.4985	0.119*	
C54	1.1151 (9)	0.1486 (7)	0.4724 (4)	0.074 (2)	
H54	1.039	0.1372	0.4553	0.088*	
N1	0.6891 (6)	0.8853 (5)	0.2768 (3)	0.0576 (15)	
N2	0.4907 (5)	0.8844 (4)	0.2321 (3)	0.0546 (15)	
N3	0.8304 (5)	0.6293 (4)	0.1078 (3)	0.0515 (14)	
N4	0.8344 (6)	0.4568 (4)	0.1060 (3)	0.0549 (15)	
N5	1.1304 (6)	0.4083 (5)	0.3616 (3)	0.0549 (15)	
N6	0.9923 (5)	0.3427 (5)	0.4472 (3)	0.0543 (15)	
O1	0.8953 (4)	0.7458 (4)	0.3172 (2)	0.0539 (12)	
O2	0.9919 (5)	0.8962 (4)	0.3525 (3)	0.0744 (15)	
O3	0.9116 (4)	0.7098 (4)	0.1875 (2)	0.0517 (11)	
O4	0.8609 (5)	0.8976 (4)	0.0959 (3)	0.0662 (14)	
O5	1.0897 (4)	0.5727 (4)	0.2805 (2)	0.0533 (12)	
O6	1.3660 (5)	0.5297 (4)	0.2500 (3)	0.0701 (14)	
S1	0.65537 (17)	0.68586 (14)	0.27060 (10)	0.0546 (5)	
S2	0.89274 (18)	0.48093 (14)	0.22979 (10)	0.0528 (5)	
S3	0.85482 (18)	0.53514 (16)	0.38161 (10)	0.0602 (5)	
Co1	0.88802 (9)	0.62353 (7)	0.27666 (5)	0.0511 (3)	

### Atomic displacement parameters (Å^2^)

**Table:**

	*U*^11^	*U*^22^	*U*^33^	*U*^12^	*U*^13^	*U*^23^
C1	0.050 (4)	0.064 (5)	0.046 (4)	−0.018 (4)	−0.008 (3)	−0.015 (4)
C2	0.049 (4)	0.056 (4)	0.060 (5)	−0.010 (3)	−0.011 (3)	−0.011 (4)
C3	0.051 (4)	0.072 (5)	0.058 (5)	−0.019 (4)	−0.013 (4)	−0.015 (4)
C4	0.064 (5)	0.060 (5)	0.080 (6)	−0.017 (4)	−0.013 (4)	−0.022 (4)
C5	0.101 (7)	0.073 (6)	0.108 (8)	−0.034 (5)	−0.016 (6)	−0.033 (5)
C6	0.087 (6)	0.095 (7)	0.105 (7)	−0.046 (6)	−0.010 (5)	−0.046 (6)
C7	0.053 (4)	0.042 (4)	0.075 (5)	−0.007 (3)	−0.018 (4)	−0.017 (4)
C8	0.062 (5)	0.065 (5)	0.077 (6)	−0.011 (4)	−0.009 (4)	−0.021 (4)
C9	0.070 (5)	0.066 (5)	0.088 (7)	−0.019 (4)	0.002 (5)	−0.033 (5)
C10	0.081 (6)	0.057 (5)	0.114 (8)	−0.014 (4)	−0.036 (6)	−0.025 (6)
C11	0.088 (6)	0.058 (5)	0.086 (7)	−0.024 (4)	−0.026 (5)	−0.006 (5)
C12	0.073 (5)	0.048 (4)	0.070 (5)	−0.018 (4)	−0.019 (4)	−0.010 (4)
C13	0.049 (4)	0.047 (4)	0.068 (5)	−0.009 (3)	−0.013 (4)	−0.008 (4)
C14	0.056 (4)	0.065 (5)	0.075 (6)	−0.024 (4)	0.001 (4)	−0.017 (4)
C15	0.055 (5)	0.068 (5)	0.115 (8)	−0.022 (4)	−0.012 (5)	−0.018 (5)
C16	0.058 (5)	0.046 (4)	0.118 (8)	−0.010 (3)	−0.030 (5)	−0.016 (5)
C17	0.065 (5)	0.060 (5)	0.073 (6)	−0.006 (4)	−0.022 (4)	−0.016 (4)
C18	0.050 (4)	0.069 (5)	0.069 (5)	−0.014 (3)	−0.007 (4)	−0.026 (4)
C19	0.048 (4)	0.051 (4)	0.058 (5)	−0.013 (3)	−0.005 (3)	−0.007 (4)
C20	0.044 (4)	0.047 (4)	0.067 (5)	−0.017 (3)	−0.003 (3)	−0.009 (4)
C21	0.054 (4)	0.050 (4)	0.058 (5)	−0.019 (3)	−0.007 (3)	−0.009 (3)
C22	0.075 (5)	0.056 (5)	0.063 (5)	−0.018 (4)	−0.022 (4)	−0.008 (4)
C23	0.092 (6)	0.058 (5)	0.069 (6)	−0.011 (4)	−0.022 (5)	0.001 (4)
C24	0.088 (6)	0.059 (5)	0.063 (5)	−0.024 (4)	−0.007 (4)	−0.003 (4)
C25	0.058 (4)	0.054 (4)	0.052 (4)	−0.019 (3)	−0.014 (3)	−0.011 (3)
C26	0.054 (4)	0.074 (5)	0.066 (5)	−0.013 (4)	−0.011 (4)	−0.018 (4)
C27	0.053 (4)	0.083 (6)	0.060 (5)	−0.012 (4)	−0.010 (4)	−0.020 (4)
C28	0.071 (5)	0.073 (5)	0.065 (5)	−0.030 (4)	−0.014 (4)	−0.010 (4)
C29	0.060 (4)	0.062 (5)	0.063 (5)	−0.016 (4)	−0.002 (4)	−0.024 (4)
C30	0.053 (4)	0.068 (5)	0.054 (5)	−0.016 (3)	−0.007 (4)	−0.020 (4)
C31	0.063 (4)	0.048 (4)	0.057 (5)	−0.016 (3)	−0.013 (4)	−0.013 (3)
C32	0.056 (4)	0.058 (5)	0.071 (5)	−0.022 (4)	−0.013 (4)	−0.013 (4)
C33	0.075 (5)	0.067 (5)	0.072 (6)	−0.029 (4)	−0.007 (4)	−0.014 (4)
C34	0.081 (5)	0.049 (4)	0.075 (6)	−0.019 (4)	−0.007 (4)	−0.016 (4)
C35	0.062 (5)	0.050 (4)	0.079 (6)	−0.001 (4)	−0.017 (4)	−0.006 (4)
C36	0.057 (4)	0.052 (4)	0.067 (5)	−0.011 (3)	−0.012 (4)	−0.008 (4)
C37	0.047 (4)	0.058 (4)	0.055 (4)	−0.022 (3)	−0.003 (3)	−0.018 (4)
C38	0.043 (4)	0.064 (5)	0.059 (5)	−0.018 (3)	−0.008 (3)	−0.015 (4)
C39	0.057 (4)	0.054 (4)	0.047 (4)	−0.020 (3)	0.003 (3)	−0.012 (3)
C40	0.062 (5)	0.058 (5)	0.060 (5)	−0.014 (4)	−0.019 (4)	−0.008 (4)
C41	0.043 (4)	0.100 (7)	0.098 (7)	−0.004 (4)	−0.019 (5)	−0.037 (6)
C42	0.044 (4)	0.113 (7)	0.089 (7)	−0.027 (5)	0.005 (4)	−0.052 (6)
C43	0.054 (4)	0.065 (5)	0.054 (5)	−0.025 (3)	0.002 (4)	−0.007 (4)
C44	0.063 (5)	0.073 (5)	0.055 (5)	−0.027 (4)	0.005 (4)	−0.013 (4)
C45	0.089 (6)	0.078 (5)	0.051 (5)	−0.033 (5)	0.008 (4)	−0.025 (4)
C46	0.066 (5)	0.100 (7)	0.081 (7)	−0.034 (5)	0.006 (5)	−0.021 (5)
C47	0.059 (5)	0.136 (9)	0.084 (7)	−0.050 (5)	0.001 (5)	−0.022 (6)
C48	0.069 (5)	0.116 (7)	0.070 (6)	−0.043 (5)	−0.012 (4)	−0.029 (5)
C49	0.057 (4)	0.051 (4)	0.061 (5)	−0.014 (3)	−0.007 (4)	−0.011 (4)
C50	0.065 (5)	0.069 (5)	0.070 (5)	−0.012 (4)	−0.011 (4)	−0.012 (4)
C51	0.061 (5)	0.103 (8)	0.080 (6)	−0.008 (5)	−0.022 (4)	0.004 (6)
C52	0.079 (7)	0.090 (8)	0.091 (8)	0.010 (6)	0.010 (6)	0.004 (6)
C53	0.114 (8)	0.066 (6)	0.090 (7)	0.006 (6)	0.000 (6)	−0.008 (5)
C54	0.082 (6)	0.070 (5)	0.070 (6)	−0.022 (5)	−0.005 (4)	−0.015 (4)
N1	0.046 (3)	0.058 (4)	0.071 (4)	−0.012 (3)	−0.014 (3)	−0.015 (3)
N2	0.042 (3)	0.056 (4)	0.072 (4)	−0.016 (3)	−0.015 (3)	−0.017 (3)
N3	0.052 (3)	0.048 (3)	0.059 (4)	−0.016 (3)	−0.012 (3)	−0.013 (3)
N4	0.060 (3)	0.046 (3)	0.062 (4)	−0.018 (3)	−0.011 (3)	−0.008 (3)
N5	0.054 (3)	0.054 (3)	0.057 (4)	−0.017 (3)	−0.009 (3)	−0.008 (3)
N6	0.048 (3)	0.059 (4)	0.057 (4)	−0.017 (3)	−0.008 (3)	−0.005 (3)
O1	0.045 (2)	0.057 (3)	0.061 (3)	−0.011 (2)	−0.012 (2)	−0.016 (2)
O2	0.062 (3)	0.082 (4)	0.089 (4)	−0.021 (3)	−0.010 (3)	−0.038 (3)
O3	0.045 (2)	0.058 (3)	0.055 (3)	−0.015 (2)	−0.009 (2)	−0.014 (2)
O4	0.073 (3)	0.058 (3)	0.072 (4)	−0.024 (3)	−0.012 (3)	−0.010 (3)
O5	0.051 (3)	0.056 (3)	0.054 (3)	−0.013 (2)	−0.010 (2)	−0.011 (2)
O6	0.059 (3)	0.081 (4)	0.077 (4)	−0.026 (3)	0.000 (3)	−0.023 (3)
S1	0.0444 (9)	0.0532 (10)	0.0670 (12)	−0.0134 (8)	−0.0088 (8)	−0.0113 (9)
S2	0.0522 (10)	0.0526 (10)	0.0546 (11)	−0.0147 (8)	−0.0077 (8)	−0.0111 (8)
S3	0.0510 (10)	0.0651 (12)	0.0587 (12)	−0.0097 (9)	−0.0024 (9)	−0.0108 (9)
Co1	0.0441 (5)	0.0536 (6)	0.0563 (6)	−0.0120 (4)	−0.0081 (4)	−0.0130 (5)

### Geometric parameters (Å, °)

**Table:**

C1—O1	1.268 (8)	C28—H28	0.93
C1—N1	1.335 (8)	C29—C30	1.378 (9)
C1—C3	1.468 (10)	C29—H29	0.93
C2—N1	1.343 (8)	C30—H30	0.93
C2—N2	1.361 (8)	C31—C36	1.381 (9)
C2—S1	1.714 (7)	C31—C32	1.383 (9)
C3—C4	1.344 (10)	C31—N4	1.423 (8)
C3—O2	1.371 (8)	C32—C33	1.376 (10)
C4—C5	1.402 (11)	C32—H32	0.93
C4—H4	0.93	C33—C34	1.379 (10)
C5—C6	1.344 (12)	C33—H33	0.93
C5—H5	0.93	C34—C35	1.383 (10)
C6—O2	1.400 (9)	C34—H34	0.93
C6—H6	0.93	C35—C36	1.379 (9)
C7—C12	1.356 (10)	C35—H35	0.93
C7—C8	1.370 (10)	C36—H36	0.93
C7—N2	1.451 (8)	C37—O5	1.263 (7)
C8—C9	1.378 (10)	C37—N5	1.340 (8)
C8—H8	0.93	C37—C39	1.470 (9)
C9—C10	1.361 (11)	C38—N5	1.335 (8)
C9—H9	0.93	C38—N6	1.362 (8)
C10—C11	1.384 (12)	C38—S3	1.710 (7)
C10—H10	0.93	C39—C40	1.342 (9)
C11—C12	1.382 (10)	C39—O6	1.361 (8)
C11—H11	0.93	C40—C41	1.434 (11)
C12—H12	0.93	C40—H40	0.93
C13—C18	1.356 (10)	C41—C42	1.333 (11)
C13—C14	1.377 (9)	C41—H41	0.93
C13—N2	1.445 (8)	C42—O6	1.362 (8)
C14—C15	1.368 (10)	C42—H42	0.93
C14—H14	0.93	C43—C44	1.383 (10)
C15—C16	1.380 (12)	C43—C48	1.399 (9)
C15—H15	0.93	C43—N6	1.437 (8)
C16—C17	1.361 (11)	C44—C45	1.378 (10)
C16—H16	0.93	C44—H44	0.93
C17—C18	1.382 (10)	C45—C46	1.367 (10)
C17—H17	0.93	C45—H45	0.93
C18—H18	0.93	C46—C47	1.362 (11)
C19—O3	1.263 (8)	C46—H46	0.93
C19—N3	1.355 (9)	C47—C48	1.361 (11)
C19—C21	1.458 (9)	C47—H47	0.93
C20—N3	1.336 (8)	C48—H48	0.93
C20—N4	1.370 (8)	C49—C50	1.362 (10)
C20—S2	1.712 (7)	C49—C54	1.376 (10)
C21—C22	1.336 (9)	C49—N6	1.458 (8)
C21—O4	1.371 (8)	C50—C51	1.397 (10)
C22—C23	1.423 (10)	C50—H50	0.93
C22—H22	0.93	C51—C52	1.356 (13)
C23—C24	1.320 (11)	C51—H51	0.93
C23—H23	0.93	C52—C53	1.379 (14)
C24—O4	1.356 (8)	C52—H52	0.93
C24—H24	0.93	C53—C54	1.388 (14)
C25—C30	1.360 (9)	C53—H53	0.93
C25—C26	1.402 (9)	C54—H54	0.93
C25—N4	1.444 (8)	O1—Co1	1.939 (5)
C26—C27	1.387 (9)	O3—Co1	1.920 (5)
C26—H26	0.93	O5—Co1	1.919 (4)
C27—C28	1.372 (10)	S1—Co1	2.217 (2)
C27—H27	0.93	S2—Co1	2.214 (2)
C28—C29	1.366 (10)	S3—Co1	2.196 (2)
			
O1—C1—N1	131.6 (7)	C33—C34—C35	119.3 (7)
O1—C1—C3	116.8 (6)	C33—C34—H34	120.3
N1—C1—C3	111.5 (6)	C35—C34—H34	120.3
N1—C2—N2	113.6 (6)	C36—C35—C34	120.1 (7)
N1—C2—S1	130.0 (5)	C36—C35—H35	120
N2—C2—S1	116.5 (5)	C34—C35—H35	120
C4—C3—O2	110.6 (7)	C35—C36—C31	120.4 (7)
C4—C3—C1	131.4 (7)	C35—C36—H36	119.8
O2—C3—C1	117.9 (6)	C31—C36—H36	119.8
C3—C4—C5	107.1 (7)	O5—C37—N5	131.3 (6)
C3—C4—H4	126.4	O5—C37—C39	116.2 (6)
C5—C4—H4	126.4	N5—C37—C39	112.4 (6)
C6—C5—C4	107.5 (8)	N5—C38—N6	114.3 (6)
C6—C5—H5	126.3	N5—C38—S3	129.0 (6)
C4—C5—H5	126.3	N6—C38—S3	116.7 (5)
C5—C6—O2	109.5 (7)	C40—C39—O6	111.4 (6)
C5—C6—H6	125.2	C40—C39—C37	130.9 (7)
O2—C6—H6	125.2	O6—C39—C37	117.6 (6)
C12—C7—C8	121.8 (7)	C39—C40—C41	104.7 (7)
C12—C7—N2	118.2 (7)	C39—C40—H40	127.6
C8—C7—N2	120.0 (7)	C41—C40—H40	127.6
C7—C8—C9	119.5 (8)	C42—C41—C40	107.6 (7)
C7—C8—H8	120.2	C42—C41—H41	126.2
C9—C8—H8	120.2	C40—C41—H41	126.2
C10—C9—C8	120.1 (9)	C41—C42—O6	110.0 (8)
C10—C9—H9	119.9	C41—C42—H42	125
C8—C9—H9	119.9	O6—C42—H42	125
C9—C10—C11	119.3 (8)	C44—C43—C48	119.8 (7)
C9—C10—H10	120.3	C44—C43—N6	118.9 (6)
C11—C10—H10	120.3	C48—C43—N6	121.3 (7)
C12—C11—C10	121.0 (8)	C45—C44—C43	118.7 (7)
C12—C11—H11	119.5	C45—C44—H44	120.6
C10—C11—H11	119.5	C43—C44—H44	120.6
C7—C12—C11	118.2 (8)	C46—C45—C44	121.3 (8)
C7—C12—H12	120.9	C46—C45—H45	119.3
C11—C12—H12	120.9	C44—C45—H45	119.3
C18—C13—C14	120.7 (7)	C47—C46—C45	119.5 (8)
C18—C13—N2	120.1 (7)	C47—C46—H46	120.2
C14—C13—N2	118.8 (7)	C45—C46—H46	120.2
C15—C14—C13	120.4 (8)	C48—C47—C46	121.2 (8)
C15—C14—H14	119.8	C48—C47—H47	119.4
C13—C14—H14	119.8	C46—C47—H47	119.4
C14—C15—C16	119.2 (8)	C47—C48—C43	119.4 (8)
C14—C15—H15	120.4	C47—C48—H48	120.3
C16—C15—H15	120.4	C43—C48—H48	120.3
C17—C16—C15	119.7 (8)	C50—C49—C54	120.5 (7)
C17—C16—H16	120.1	C50—C49—N6	121.4 (7)
C15—C16—H16	120.1	C54—C49—N6	118.1 (7)
C16—C17—C18	121.2 (8)	C49—C50—C51	119.0 (8)
C16—C17—H17	119.4	C49—C50—H50	120.5
C18—C17—H17	119.4	C51—C50—H50	120.5
C13—C18—C17	118.7 (7)	C52—C51—C50	120.0 (9)
C13—C18—H18	120.6	C52—C51—H51	120
C17—C18—H18	120.6	C50—C51—H51	120
O3—C19—N3	129.7 (6)	C51—C52—C53	121.9 (9)
O3—C19—C21	117.7 (6)	C51—C52—H52	119.1
N3—C19—C21	112.6 (6)	C53—C52—H52	119.1
N3—C20—N4	113.4 (6)	C52—C53—C54	117.5 (10)
N3—C20—S2	129.6 (6)	C52—C53—H53	121.2
N4—C20—S2	116.8 (5)	C54—C53—H53	121.2
C22—C21—O4	110.2 (6)	C49—C54—C53	121.1 (9)
C22—C21—C19	131.5 (7)	C49—C54—H54	119.5
O4—C21—C19	118.3 (6)	C53—C54—H54	119.5
C21—C22—C23	106.3 (7)	C1—N1—C2	123.2 (6)
C21—C22—H22	126.9	C2—N2—C13	123.4 (6)
C23—C22—H22	126.9	C2—N2—C7	120.3 (5)
C24—C23—C22	106.6 (7)	C13—N2—C7	116.2 (5)
C24—C23—H23	126.7	C20—N3—C19	123.9 (6)
C22—C23—H23	126.7	C20—N4—C31	123.1 (6)
C23—C24—O4	111.3 (7)	C20—N4—C25	121.3 (5)
C23—C24—H24	124.4	C31—N4—C25	115.6 (6)
O4—C24—H24	124.4	C38—N5—C37	123.1 (6)
C30—C25—C26	120.5 (6)	C38—N6—C43	121.9 (6)
C30—C25—N4	121.1 (6)	C38—N6—C49	121.9 (6)
C26—C25—N4	118.3 (7)	C43—N6—C49	116.0 (6)
C27—C26—C25	117.8 (7)	C1—O1—Co1	126.7 (4)
C27—C26—H26	121.1	C3—O2—C6	105.2 (6)
C25—C26—H26	121.1	C19—O3—Co1	127.0 (4)
C28—C27—C26	121.7 (7)	C24—O4—C21	105.7 (6)
C28—C27—H27	119.2	C37—O5—Co1	127.7 (4)
C26—C27—H27	119.2	C39—O6—C42	106.3 (6)
C29—C28—C27	119.1 (7)	C2—S1—Co1	104.5 (2)
C29—C28—H28	120.4	C20—S2—Co1	106.4 (2)
C27—C28—H28	120.4	C38—S3—Co1	106.0 (3)
C28—C29—C30	120.8 (7)	O5—Co1—O3	88.20 (19)
C28—C29—H29	119.6	O5—Co1—O1	85.77 (19)
C30—C29—H29	119.6	O3—Co1—O1	85.5 (2)
C25—C30—C29	120.2 (7)	O5—Co1—S3	93.19 (15)
C25—C30—H30	119.9	O3—Co1—S3	176.11 (15)
C29—C30—H30	119.9	O1—Co1—S3	90.95 (15)
C36—C31—C32	119.5 (6)	O5—Co1—S2	91.05 (15)
C36—C31—N4	121.9 (6)	O3—Co1—S2	93.98 (15)
C32—C31—N4	118.4 (6)	O1—Co1—S2	176.80 (14)
C33—C32—C31	120.0 (7)	S3—Co1—S2	89.62 (8)
C33—C32—H32	120	O5—Co1—S1	178.62 (16)
C31—C32—H32	120	O3—Co1—S1	90.58 (13)
C32—C33—C34	120.7 (7)	O1—Co1—S1	93.51 (14)
C32—C33—H33	119.6	S3—Co1—S1	87.99 (7)
C34—C33—H33	119.6	S2—Co1—S1	89.66 (7)
			
O1—C1—C3—C4	−179.4 (8)	C14—C13—N2—C7	−70.9 (8)
N1—C1—C3—C4	1.9 (12)	C12—C7—N2—C2	105.8 (8)
O1—C1—C3—O2	3.8 (10)	C8—C7—N2—C2	−76.9 (9)
N1—C1—C3—O2	−174.9 (6)	C12—C7—N2—C13	−71.2 (8)
O2—C3—C4—C5	−0.3 (10)	C8—C7—N2—C13	106.1 (8)
C1—C3—C4—C5	−177.3 (8)	N4—C20—N3—C19	−169.6 (6)
C3—C4—C5—C6	0.6 (11)	S2—C20—N3—C19	14.7 (10)
C4—C5—C6—O2	−0.6 (11)	O3—C19—N3—C20	−4.5 (11)
C12—C7—C8—C9	1.0 (11)	C21—C19—N3—C20	175.4 (6)
N2—C7—C8—C9	−176.2 (6)	N3—C20—N4—C31	172.6 (6)
C7—C8—C9—C10	−0.1 (12)	S2—C20—N4—C31	−11.1 (9)
C8—C9—C10—C11	−0.6 (12)	N3—C20—N4—C25	−7.0 (9)
C9—C10—C11—C12	0.5 (12)	S2—C20—N4—C25	169.3 (5)
C8—C7—C12—C11	−1.1 (11)	C36—C31—N4—C20	−63.5 (10)
N2—C7—C12—C11	176.2 (6)	C32—C31—N4—C20	121.4 (7)
C10—C11—C12—C7	0.3 (12)	C36—C31—N4—C25	116.1 (7)
C18—C13—C14—C15	1.4 (11)	C32—C31—N4—C25	−59.0 (9)
N2—C13—C14—C15	173.9 (6)	C30—C25—N4—C20	121.4 (8)
C13—C14—C15—C16	−1.8 (11)	C26—C25—N4—C20	−63.8 (9)
C14—C15—C16—C17	1.2 (11)	C30—C25—N4—C31	−58.2 (9)
C15—C16—C17—C18	−0.3 (11)	C26—C25—N4—C31	116.6 (7)
C14—C13—C18—C17	−0.5 (11)	N6—C38—N5—C37	−173.5 (6)
N2—C13—C18—C17	−172.9 (6)	S3—C38—N5—C37	3.9 (11)
C16—C17—C18—C13	−0.1 (11)	O5—C37—N5—C38	−12.4 (12)
O3—C19—C21—C22	178.2 (8)	C39—C37—N5—C38	165.3 (6)
N3—C19—C21—C22	−1.8 (11)	N5—C38—N6—C43	−176.6 (6)
O3—C19—C21—O4	−2.7 (9)	S3—C38—N6—C43	5.7 (9)
N3—C19—C21—O4	177.4 (6)	N5—C38—N6—C49	8.5 (10)
O4—C21—C22—C23	0.3 (9)	S3—C38—N6—C49	−169.2 (5)
C19—C21—C22—C23	179.5 (7)	C44—C43—N6—C38	−117.6 (8)
C21—C22—C23—C24	0.6 (9)	C48—C43—N6—C38	64.3 (10)
C22—C23—C24—O4	−1.2 (10)	C44—C43—N6—C49	57.6 (9)
C30—C25—C26—C27	0.2 (11)	C48—C43—N6—C49	−120.5 (8)
N4—C25—C26—C27	−174.6 (7)	C50—C49—N6—C38	57.1 (10)
C25—C26—C27—C28	0.1 (12)	C54—C49—N6—C38	−125.7 (8)
C26—C27—C28—C29	0.4 (12)	C50—C49—N6—C43	−118.1 (8)
C27—C28—C29—C30	−1.1 (12)	C54—C49—N6—C43	59.1 (9)
C26—C25—C30—C29	−1.0 (11)	N1—C1—O1—Co1	14.3 (11)
N4—C25—C30—C29	173.7 (6)	C3—C1—O1—Co1	−164.0 (5)
C28—C29—C30—C25	1.4 (11)	C4—C3—O2—C6	−0.1 (9)
C36—C31—C32—C33	−1.1 (11)	C1—C3—O2—C6	177.4 (7)
N4—C31—C32—C33	174.2 (7)	C5—C6—O2—C3	0.5 (10)
C31—C32—C33—C34	1.8 (12)	N3—C19—O3—Co1	−26.9 (10)
C32—C33—C34—C35	−0.7 (12)	C21—C19—O3—Co1	153.2 (5)
C33—C34—C35—C36	−1.1 (12)	C23—C24—O4—C21	1.4 (9)
C34—C35—C36—C31	1.8 (12)	C22—C21—O4—C24	−1.0 (8)
C32—C31—C36—C35	−0.7 (11)	C19—C21—O4—C24	179.6 (6)
N4—C31—C36—C35	−175.8 (7)	N5—C37—O5—Co1	−11.8 (11)
O5—C37—C39—C40	178.2 (7)	C39—C37—O5—Co1	170.6 (4)
N5—C37—C39—C40	0.1 (11)	C40—C39—O6—C42	0.4 (8)
O5—C37—C39—O6	−1.3 (9)	C37—C39—O6—C42	179.9 (6)
N5—C37—C39—O6	−179.3 (6)	C41—C42—O6—C39	0.2 (9)
O6—C39—C40—C41	−0.7 (9)	N1—C2—S1—Co1	−22.5 (8)
C37—C39—C40—C41	179.8 (7)	N2—C2—S1—Co1	158.8 (5)
C39—C40—C41—C42	0.8 (9)	N3—C20—S2—Co1	2.9 (7)
C40—C41—C42—O6	−0.6 (10)	N4—C20—S2—Co1	−172.7 (4)
C48—C43—C44—C45	−0.8 (11)	N5—C38—S3—Co1	19.4 (7)
N6—C43—C44—C45	−178.9 (7)	N6—C38—S3—Co1	−163.3 (5)
C43—C44—C45—C46	−0.6 (12)	C37—O5—Co1—O3	−153.8 (6)
C44—C45—C46—C47	1.1 (14)	C37—O5—Co1—O1	120.6 (6)
C45—C46—C47—C48	−0.2 (15)	C37—O5—Co1—S3	29.9 (5)
C46—C47—C48—C43	−1.2 (14)	C37—O5—Co1—S2	−59.8 (5)
C44—C43—C48—C47	1.7 (13)	C19—O3—Co1—O5	125.6 (5)
N6—C43—C48—C47	179.8 (7)	C19—O3—Co1—O1	−148.5 (5)
C54—C49—C50—C51	−1.5 (12)	C19—O3—Co1—S2	34.7 (5)
N6—C49—C50—C51	175.6 (7)	C19—O3—Co1—S1	−55.0 (5)
C49—C50—C51—C52	0.7 (13)	C1—O1—Co1—O5	147.3 (6)
C50—C51—C52—C53	0.9 (15)	C1—O1—Co1—O3	58.8 (6)
C51—C52—C53—C54	−1.6 (15)	C1—O1—Co1—S3	−119.6 (5)
C50—C49—C54—C53	0.7 (13)	C1—O1—Co1—S1	−31.6 (6)
N6—C49—C54—C53	−176.5 (8)	C38—S3—Co1—O5	−27.9 (3)
C52—C53—C54—C49	0.8 (14)	C38—S3—Co1—O1	−113.7 (3)
O1—C1—N1—C2	8.9 (12)	C38—S3—Co1—S2	63.1 (3)
C3—C1—N1—C2	−172.6 (6)	C38—S3—Co1—S1	152.8 (3)
N2—C2—N1—C1	178.9 (6)	C20—S2—Co1—O5	−108.4 (3)
S1—C2—N1—C1	0.1 (11)	C20—S2—Co1—O3	−20.2 (3)
N1—C2—N2—C13	169.1 (6)	C20—S2—Co1—S3	158.4 (2)
S1—C2—N2—C13	−11.9 (9)	C20—S2—Co1—S1	70.4 (2)
N1—C2—N2—C7	−7.6 (10)	C2—S1—Co1—O3	−56.2 (3)
S1—C2—N2—C7	171.3 (5)	C2—S1—Co1—O1	29.4 (3)
C18—C13—N2—C2	−75.3 (9)	C2—S1—Co1—S3	120.2 (3)
C14—C13—N2—C2	112.2 (8)	C2—S1—Co1—S2	−150.2 (3)
C18—C13—N2—C7	101.6 (8)		
